# Chemical Identification in the Specular and Off-Specular Rough-Surface Scattered Terahertz Spectra Using Wavelet Shrinkage

**DOI:** 10.1109/access.2021.3059424

**Published:** 2021-02-15

**Authors:** MAHMOUD E. KHANI, M. HASSAN ARBAB

**Affiliations:** Department of Biomedical Engineering, Stony Brook University, Stony Brook, NY 11794, USA

**Keywords:** Chemical identification, maximal overlap discrete wavelet transform (MODWT), phase function effects, pyramid algorithm, reflection-mode spectroscopy, rough surface scattering, terahertz time-domain spectroscopy (THz-TDS), wavelet shrinkage, off-specular scattered spectra

## Abstract

We present the development and implementation of a novel wavelet shrinkage technique for the retrieval of obscured characteristic resonant signatures in the scattered terahertz (THz) reflectivity of molecular crystals. In this implementation, the wavelet basis functions associated with the absorption features were identified using the second-order total variation of the wavelet coefficients. Additionally, wavelet coefficients at certain scales were modified using the phase function corrections and wavelet hard thresholding. Reconstruction of the original spectra using these modified wavelet coefficients yielded the exact resonant frequencies of the chemicals, which were otherwise unrecognizable in the spectral artifacts of the rough surface scattering. We examined the robustness of this method over controlled levels of rough surface scattering, validated using the Kirchhoff approximation, in spectroscopic targets made from *α*-lactose monohydrate and 4-aminobenzoic acid (PABA), which have close spectral lines. We successfully retrieved the spectral absorption fingerprints in both specular and off-specular reflection geometries. This technique can be utilized for stand-off material characterization using the THz reflection spectroscopy in uncontrolled environments and potentially can be adopted for other broadband spectroscopic modalities.

## 1. INTRODUCTION

A broadband terahertz (THz) pulse can resolve the low-frequency vibrational and rotational modes of molecular crystals [1]. These molecular motions, which are associated with intra- or inter-molecular interactions, such as the weak hydrogen bonds or the crystalline lattice modes, appear as resonant signatures in the dielectric functions measured using THz time-domain spectroscopy (THz-TDS) [2]. Therefore, they can be utilized as characteristic spectral fingerprints for material characterization [3], [4]. However, chemical identification using transmission spectroscopy geometries is often met with practical challenges in real-world applications, such as the lack of access to the transmitted signal for remote sensing or the signal attenuation in highly-absorptive materials. Therefore, reflection-mode spectroscopy is preferable for many nondestructive testing and biomedical spectroscopy applications [5]–[7]. However, in the reflection-mode THz spectroscopy, surface height variations on the order of the illumination wavelengths result in significant rough surface scattering, which can distort or obscure the resonant signatures [8]–[10]. Although it may reduce the signal-to-noise ratio (SNR) and cause spectral signal distortions, rough surface scattering would allow for flexible emitter-detector geometries. For example, it can enable spectroscopic measurements using the back-scattered beam towards a collocated emitter-detector setup [11], [12]. On the other hand, random variations in a sample’s surface height result in a random change in the phase of the THz fields reflected from that sample as compared to a perfect reflector, which is often used as a reference for the Fourier-domain deconvolution, causing additional phase ambiguity in the extracted dielectric functions [13], [14]. Although rigorous experimental and computational techniques have been employed to avoid the phase error in reflection THz-TDS [14]–[19], they have not been proved effective in the presence of rough surface scattering, where the phase error is often a random variable [20], [21]. However, despite the phase ambiguity problem, the local maxima in the derivative of THz reflectivity with respect to frequency can still reveal the center frequencies of a substance’s resonant modes [5], [6]. Nonetheless, due to the rough surface scattering, artifacts associated with random noise and scattering effects can dominate the derivative of THz reflectivity spectrum, masking the characteristic resonant signatures of the materials [21]–[23].

The appearance of these scattering-induced spectral artifacts has encouraged development of computational techniques to distinguish between them and characteristic spectral fingerprints. [21], [23]–[30]. Cepstrum, anagram of spectrum, filtering, i.e. the low-pass or band-pass filtering of the Fourier-domain THz spectra, has been shown effective for identification of resonant signatures in the derivative of THz scattered reflectivity [21]. However, this type of analysis requires designing material-specific cepstrum-domain filters, limiting its robustness for standoff detection of unknown chemicals. Spectral dynamics analysis using integral correlation criteria has also been utilized for identification of rough-surface materials using THz reflection spectroscopy [27]. This technique relies on acquiring multiple internal reflections, which would not be available in studying single-layer semi-infinite solids. Wavelet transforms have also been proved useful for chemical recognition using the THz-TDS. However, previous work has been limited to measurements in the specular directions only [23], [28]–[31]. Importantly, THz spectroscopy in scattering-mitigated off-specular geometries will enable stand-off detection applications in arbitrary detection angles, which has not been possible so far due to the afore-mentioned spectral artifacts. For instance, by increasing the surface roughness, the increase in scattering-induced artifacts in the wavelet domain diminishes the ability of wavelets for identifying the resonant signatures in previous algorithms [23], [28], [30].

In this paper, we demonstrate the implementation of a new wavelet-domain computational technique, i.e., the wavelet shrinkage scheme, for the identification of the exact characteristic resonant frequencies of molecular crystals from the scattered THz reflectivities in both specular and off-specular angles. In this approach, using the second-order total variation of the wavelet coefficients, we identify the wavelet basis functions that capture a material’s resonant signatures. We further modify the wavelet coefficients generated by these wavelet bases using a decomposition level-based hard thresholding technique. We also discuss the phase function corrections required for the proper threshold selection in the maximal overlap discrete wavelet transform (MODWT). We examine the robustness of this technique over sample disks made from *α*-lactose monohydrate and 4-aminobenzoic acid (PABA), onto which controlled levels of surface roughness were applied and validated by the Kirchhoff approximation model. We show that the wavelet shrinkage technique enables identification of resonant frequencies obscured by the rough surface scattering in both specular and off-specular detection geometries.

The previous wavelet methods were limited to the identification in a specular direction. This limitation is caused by using individual wavelet coefficients in the wavelet domain for spectral analysis. In contrast, this paper introduces a new methodology to reconstruct the extinction spectra back in the frequency domain using the wavelet shrinkage algorithm, which importantly utilizes all selected wavelet coefficients simultaneously. We show that this key distinction will allow for off-specular detection of chemicals for the very first time in the THz regime. Also, previous work has been focused on the retrieval of the *α*-lactose’s resonant mode at 0.53 THz [23]. Here, we demonstrate the robustness and utility of the new wavelet shrinkage technique in identification of other spectral resonances in different materials having close and overlapping spectral lines. Significantly, the approach presented here does not rely on any *a priori* information about the sample materials’ dielectric functions (spectral fingerprints) or the characteristics of the rough surface scattering. In addition, it does not utilize averaging over a multitude of surface realizations or multiple internal reflections. Moreover, it yields the exact spectral positions of the resonant frequencies at both lower and higher ends of the spectrum, and the reconstructed spectra are free of any noise- or scattering-associated artifacts. Finally, we will discuss the bandwidth limitations of the proposed technique as the surface roughness increases, or the scattered energy diminishes in higher detection angles.

## II. MATERIALS AND METHODS

### A. SPECTROSCOPIC TARGETS

**FIGURE 1.**
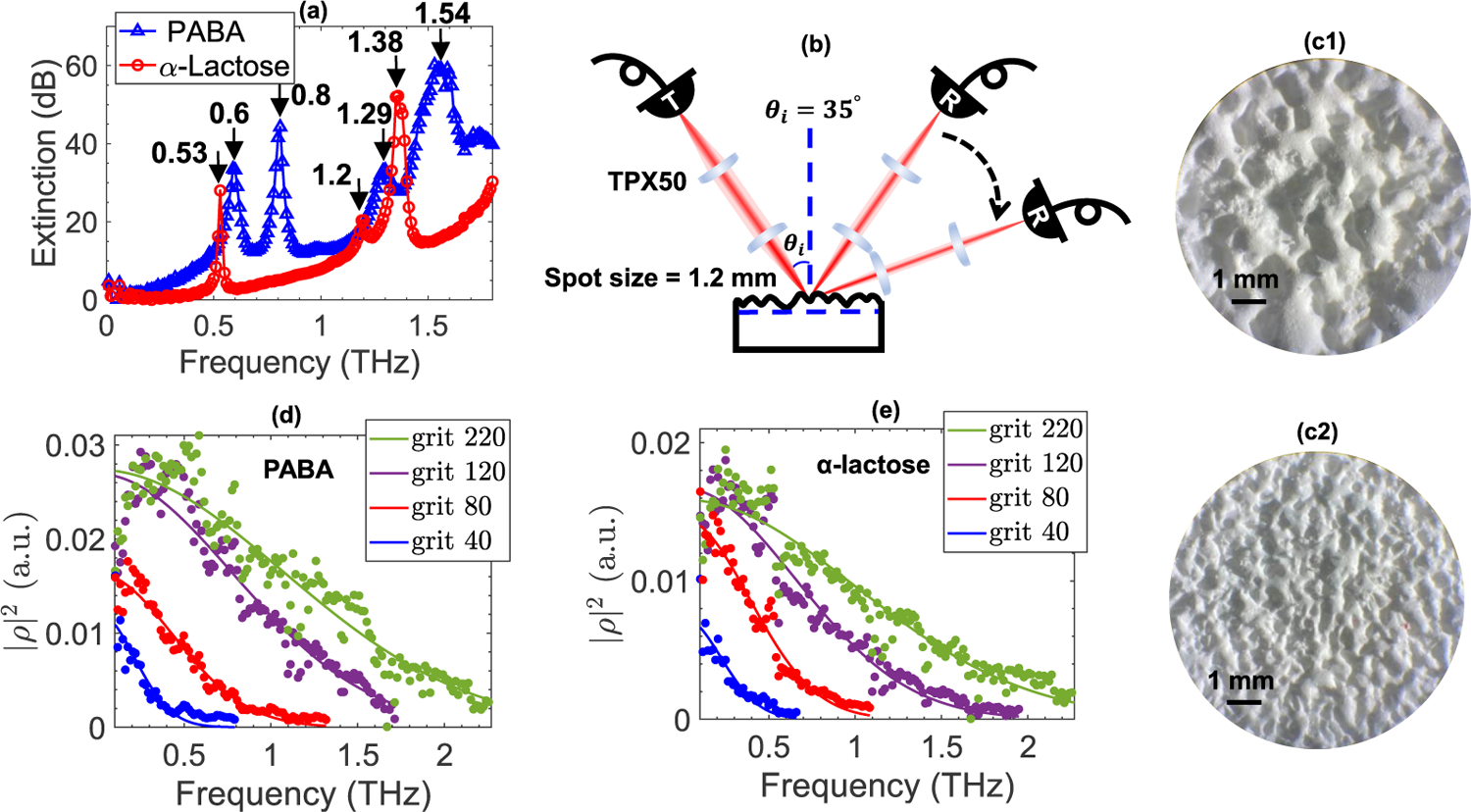
(a) The extinction spectra of *α*-lactose monohydrate and 4-aminobenzoic acid (PABA) measured by THz-TDS in transmission mode to exhibit the dielectric resonant signatures of the samples, (b) schematic of the measurement apparatus designed for the collection of THz radiation scattered in specular and off-specular directions, (c1-c2) microscopic images illustrating the surface of grit 40 and grit 80 rough-surface samples, (d) the specular reflectivities of sample disks made from *α*-lactose monohydrate with grit 40, 80, 120, and 220 rough surfaces, and (e) similar to (d) for PABA. In (d) and (e), the Kirchhoff approximation, given by Eq. (1), shown by the solid lines, is numerically fitted to the measurements, shown by the dotted lines.

We used sandpapers with 4 different roughness levels, including 220, 120, 80, and 40 grits (Norton Abrasives, Worcester, MA, USA), for creating controlled degrees of rough surface scattering, where the grit 40 sandpaper causes the highest degree of surface roughness. We prepared two sample disks at each roughness level. One sample was made from *α*-lactose monohydrate (Spectrum Chemical Mfg. Corp., Gardena, CA, USA) with resonant frequencies at 0.53, 1.2, and 1.38 THz, and one from 4-aminobenzoic acid (PABA) (Sigma-Aldrich Corp., St. Louis, MO, USA) with resonant frequencies at 0.6, 0.8, 1.29, and 1.54 THz. Figure 1(a) compares the extinction spectra of *α*-lactose monohydrate and PABA measured by THz-TDS in transmission mode. We mixed each chemical with ultra-fine high-density polyethylene (HDPE) (1:1 ratio), and pressed the mixture with a piece of sandpaper placed on its top surface under 3000 psi load for an approximate half hour period, yielding pellet disks with approximately 4 mm thickness and 50 mm diameter. Therefore, each sample was thick enough to avoid any overlaps between the front- and back-surface-reflected THz pulses. Figures 1(c1) and 1(c2) exhibit optical images from the surface of samples with grit 40 and grit 80 rough surface, respectively.

### B. MEASUREMENT SETUP

We used a modified TERA-SMART (Menlo Systems Inc, Newton, NJ, USA), which is a commercial THz time-domain spectrometer, for taking reflection spectroscopic measurements from the surface of each sample. In TERA-SMART, two fiber-coupled photoconductive antenna (PCA), excited by a 1560 nm femtosecond laser, in addition to a mechanically-moving delay stage carry out the generation and detection of the THz pulses. We used two TPX50 lenses with 50 mm focal length to collimate and focus the generated THz pulses on each sample’s surface. Using Similar lenses, we recollimated and refocused the reflected waves on the detector PCA. Each sample was fixed on a metallic sample holder. Monitoring the time-of-arrival for the THz pulses reflected from the front surface allowed for adjusting the sample holder to eliminate any possible tilt in the sample. The size of the Gaussian beam at focus, surface of each sample, was approximately 1.2 mm using knife-edge measurements. To measure the waves scattered to off-specular directions, we mounted the detection arm on a rotating rail, pivoting around the center of the sample to collect the reflected rays at different angles, as shown in Fig. 1(b).

### C. KIRCHHOFF APPROXIMATION OF THE ROUGH SURFACE SCATTERING

Using the surface properties of a solid material, such as the RMS surface height and the correlation length, the Kirchhoff approximation can predict the electromagnetic fields scattered in specular and off-specular directions [23], [32]–[35]. The Kirchhoff approximation treats each local facet of a rough surface as an infinitesimal segment of a smooth surface located on a tangent plane of the rough surface. Therefore, the Fresnel reflection coefficient of each smooth surface can determine the local scattered field [8]. Accordingly, the specular reflectivity in the incidence plane of a rough surface with a Gaussian height distribution undergoes a Gaussian frequency roll-off given by,

(1) $${\left|\rho \right|}^{2}\propto {\left|r\right|}^{2}\text{exp}\left(-4k^{2}\sigma^{2}cos^{2}\left(\theta_{i}\right)\right),$$

where ${\left|\rho \right|}^{2}$ is the specular reflectivity, *r* is the Fresnel reflection coefficient, *k* is the free-space wavenumber, *σ* is the RMS surface height, and *θ*_*i*_ is the incidence angle with respect to the surface normal. Therefore, the exponent term in Eq. (1), known as the Rayleigh factor, can be utilized to find the RMS surface height of a sample using its reflectivity.

Figures 1(d) and 1(e) illustrate the specular reflectivities of rough-surface samples made from *α*-lactose monohydrate and PABA, respectively. The Kirchhoff approximation was numerically fitted to each reflectivity, shown by the solid lines in Figs. 1(d–e). After calculating the RMS surface height, *σ*, from the exponent term in the fitted Kirchhoff approximation, the Fraunhofer criterion given by [8],

(2) $$\sigma \geq \frac{\lambda }{32}cos\left(\theta_{i}\right),$$

determines the wavelength threshold *λ*. The rough surface scattering will be significant for any wavelength smaller than this threshold. Table 1 compares the RMS heights obtained from the Kirchhoff approximation of the reflectivities shown in Fig. 1 with those reported in the literature for each sandpaper grit [21], [31], [36]. Noteworthy here, the RMS height of a pressed powder-made pellet can be affected by different factors, such as the pressure of the hydraulic press or the powders’ cohesion factor, and can be different from a sandpaper’s reported height. Nonetheless, values obtained from the Kirchhoff approximation serve as approximate surface roughness indicators, and together with the Fraunhofer criterion specify whether a particular resonant signature was affected by the rough surface scattering. Here, the *σ* s obtained for grit 220, 120, and 80 by the Kirchhoff approximation are very close to those reported in the literature. Only the grit 40 exhibits 42 *µ*m difference, which can be attributed to the pellet press pressure, and also the degree by which the sandpaper’s roughness was transferred to the sample surface. Nevertheless, for a 91 *µ*m RMS height, the Fraunhofer criterion indicates that all the resonant signatures above 80 GHz were affected by the electromagnetic scattering. Also note that the wavelet shrinkage technique presented in the following sections does not rely on the RMS surface heights obtained using the Kirchhoff approximation model.

**FIGURE 2.**
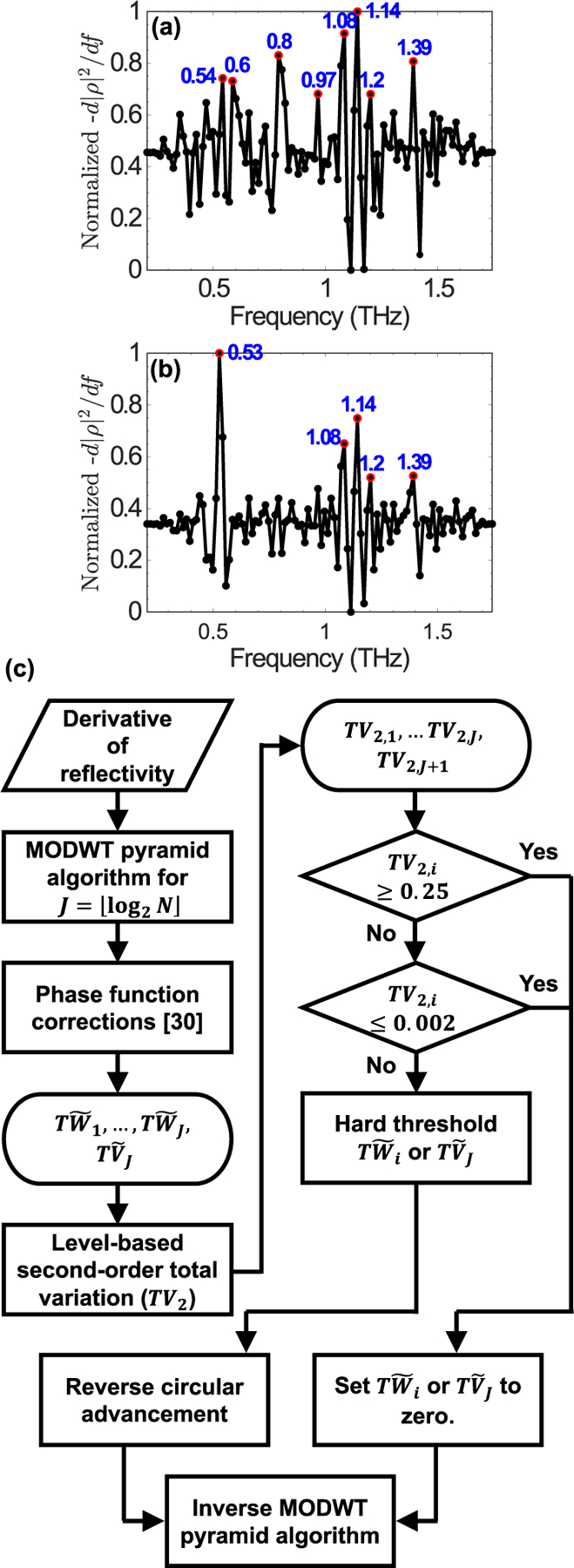
The derivative of specular reflectivity from samples made from (a) PABA and (b) *α*-lactose monohydrate with grit 220 rough surface. Peak recognition based on the height thresholding resulted in erroneous resonant frequencies, (c) flowchart representing the wavelet shrinkage of derivative of THz reflectivity for the retrieval of obscured resonant frequencies.

It can be noted that the resonant frequencies of *α*-lactose and PABA were not readily identifiable in the reflectivity spectra shown in Figs. 1(d–e). Particularly at greater roughness levels, the available bandwidth in specular direction was narrower, resulting in obscured resonant signatures. Additionally, because of the phase ambiguity in reflection spectroscopy, extinction coefficients similar to those shown in Fig. 1(a) were not attainable. In such circumstances, the negative derivative of the THz reflectivity with respect to frequency can still provide the peak positions of the absorption resonant frequencies with less than 0.02 THz deviations [5], [6], [21]. However, in the presence of scattering, identifying the resonant frequencies in the derivative of THz reflectivity is further complicated by the amplification of the noise and scattering artifacts. For example, Fig. 2(a) illustrates the derivative of THz reflectivity from a PABA sample with grit 220 rough surface. In Fig. 2(a), a peak recognition algorithm based on the peak’s height thresholding would not yield the center frequencies of the PABA resonant modes only. Among the peaks delineated in Fig. 2(a), only those at 0.6 and 0.8 THz were associated with the PABA absorption resonances, while the PABA’s higher-frequency resonances at 1.29 and 1.54 THz were obscured and masked by the scattering artifacts. Similarly, Fig. 2(b) shows the derivative of THz reflectivity from an *α*-lactose monohydrate sample with grit 220 rough surface. Likewise, only the peak marked at 0.53 THz represented a resonant frequency, while the others were either caused by the atmospheric water vapor absorption lines or the measurement noise and scattering artifacts.

### D. WAVELET SHRINKAGE

The wavelet shrinkage technique can effectively identify the characteristic resonant frequencies in the derivative spectra shown in Figs. 2(a–b). It can also recover the resonant signatures obscured by rough surface scattering, such as the PABA’s resonances at 1.29 and 1.54 THz. The flowchart in Fig. 2(c) illustrates the implementation steps. After finding the wavelet and scaling coefficients of the derivative of THz reflectivity, we correct the phase distortions caused by the wavelet and scaling filters in the decomposition stage [30]. Next, we find the decomposition levels that better capture a material’s absorption signatures, while we exclude those mainly associated with the scattering effects. Afterwards, we find an appropriate threshold at each remaining scale to further remove the scattering artifacts. Reconstruction of the original spectrum from modified wavelet and scaling coefficients yields the characteristic resonant frequencies, even those obscured by scattering. In the following, we will describe each step of the spectral reconstruction algorithm in more details.

#### 1) MAXIMAL OVERLAP DISCRETE WAVELET TRANSFORM

To calculate the wavelet, ${\tilde{W}}_{j}(f)$, and scaling coefficients, ${\tilde{V}}_{j}(f)$, as functions of frequency, *f*, we used the maximal overlap discrete wavelet transform (MODWT) pyramid algorithm, given for the *j*th level of decomposition by [37]

(3) $${\tilde{W}}_{j}\left(f\right)=\sum_{k=0}^{L-1}\tilde{h}\left(k\right){\tilde{V}}_{j-1}\left(f-2^{j-1}k\;\text{mod}\;N\right),$$

and

(4) $${\tilde{V}}_{j}\left(f\right)=\sum_{k=0}^{L-1}\tilde{g}\left(k\right){\tilde{V}}_{j-1}\left(f-2^{j-1}k\;\text{mod}\;N\right),$$

where ${\tilde{V}}_{0}\left(f\right)=-d{\left|\rho \left(f\right)\right|}^{2}/df$ and $j=1,\ldots ,\left\lfloor {\text{log}}_{2}\left(N\right)\right\rfloor$, while *N* is the size of the reflectivity spectrum,${\left|\rho \left(f\right)\right|}^{2}$. In Eqs. (3–4), $\tilde{h}\left(k\right)$ and $\tilde{g}\left(k\right)$ represent the MODWT wavelet and scaling filters with size *L*, obtained from the same mother wavelet function [37], [38], and the ‘‘mod’’ operator indicates circular convolution. Here, we used the LA(8) mother wavelet, i.e., the least asymmetric wavelet filter with eight taps, also known as ‘‘sym4’’, indicating the symlet wavelet function with four vanishing moments [39]. The four vanishing moments guarantee perfect reconstruction of the polynomials up to order three [40]. We applied MODWT for *J =* 6 levels of decomposition. The sixth-level wavelet and scaling coefficients only represented the baseline of the derivative spectrum. Therefore, calculating higher decomposition levels for extracting the resonant modes was not justified. Importantly, the required number of decomposition levels depends on the sampling interval of *ρ*(*f*), *δf*. Because the *j*th-level wavelet coefficients represent the differences of a signal’s localized averages over the scale $\sigma_{j}=\delta f\cdot 2^{j-1}$ [37], a finer *δf* necessitates using higher decomposition levels to obtain the same features extracted by lower-level wavelet coefficients at a coarser sampling.

#### 2) MODWT ZERO-PHASE PYRAMID ALGORITHM

**FIGURE 3.**
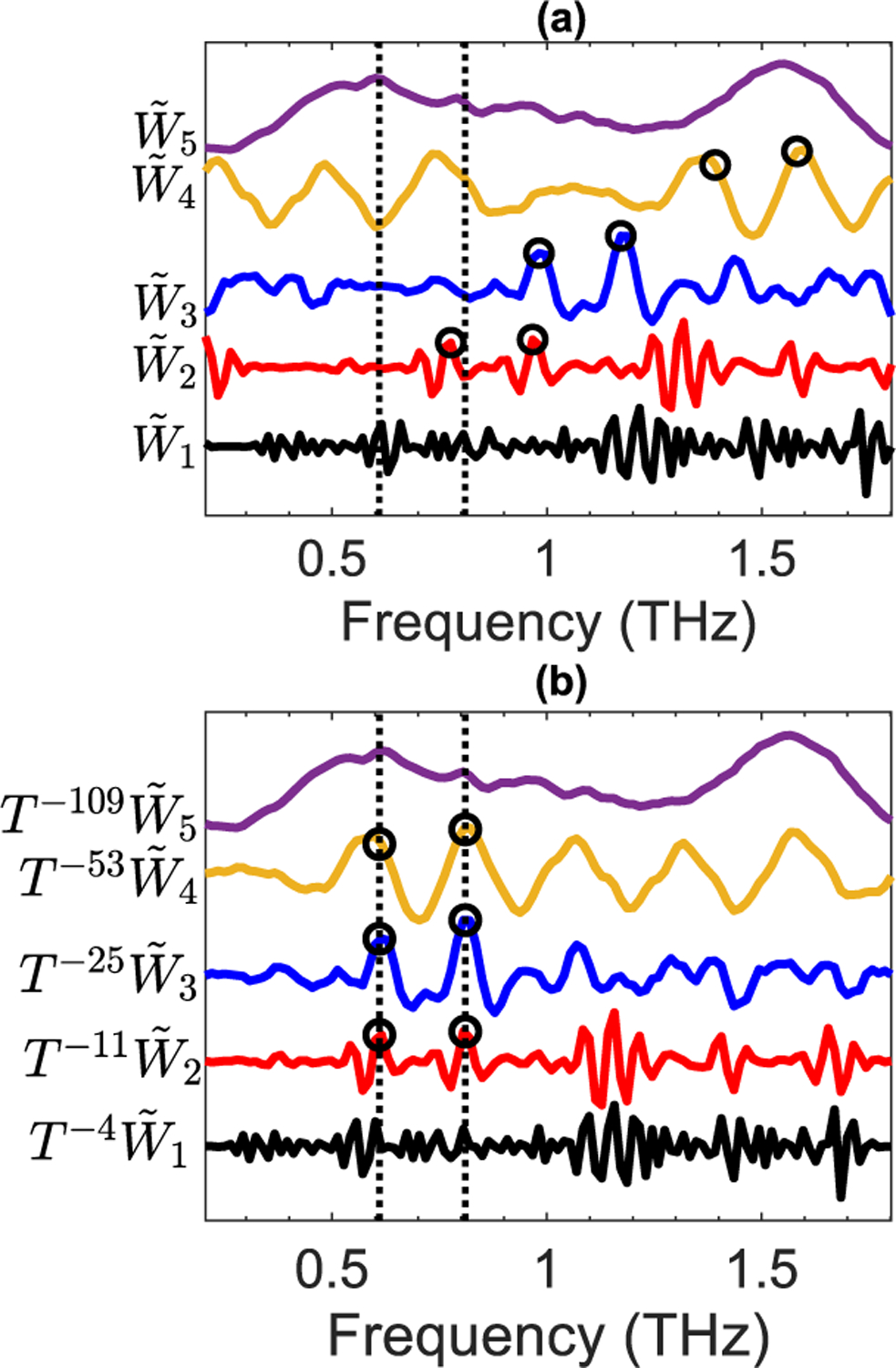
(a) The vertically-offset MODWT wavelet coefficients of the derivative of the PABA reflectivity up to the fifth level. The vertical dashed lines delineate the PABA resonant frequencies at 0.6 and 0.8 THz. Resonant features in the wavelet domain that should appear in these frequencies are delineated using the black circles in the second to fourth-level wavelet coefficients to show the misalignments between them, (b) MODWT wavelet coefficients circularly advanced to align the features in the wavelet domain with the original spectrum.

Figure 3(a) exhibits the vertically-offset wavelet coefficients of the derivative of PABA reflectivity, shown in Fig. 2(a), up to the fifth decomposition level, while the wavelet coefficients at each level were min-max normalized. Note that in Fig. 3(a), the characteristic spectral features extracted in the wavelet domain are not in alignment with those in the original derivative spectrum, whose locations at 0.6 and 0.8 THz are delineated using the vertical dashed lines. The wavelet coefficients associated with these resonant frequencies, marked by the black circles, demonstrate a decomposition level-dependent shift with respect to their correct positions. These misalignments are originated from the phase functions of the wavelet and scaling filters used in the wavelet transform’s pyramid algorithm [28], [30]. Especially when choosing the wavelet shrinkage thresholds from a specific interval at each decomposition level [41], these misalignments can lead to selecting erroneous threshold values. In Fig. 3(b), we show that by advancing the wavelet coefficients at each level by a specific number, shown as the power of *T* in $T^{-v_{j}}{\tilde{W}}_{j}$, these misalignments can get compensated, achieving an effective zero-phase MODWT pyramid algorithm. If a filter has a linear phase function given by θ(f)=2πfv, where *f* represents the normalized discrete frequency, its phase function will shift the outputs by *ν* units. Therefore, circularly shifting the outputs of such filter by *ν* units will yield an effective zero-phase filter. The orthogonal wavelet and scaling filters used in the MODWT algorithm do not demonstrate deterministic linear phase functions [37], [39]. Additionally, because in the pyramid algorithm implementation given by Eqs. (3) and (4) wavelet and scaling filters are used recursively, the misalignments caused by their phase functions become more complicated. However, among the Daubechies orthogonal wavelet filters introduced in [39], the least asymmetric wavelets demonstrate the most symmetrical shapes and therefore the highest phase function linearity. Consequently, for the LA(L) mother wavelets, where L represents the mother wavelet length, it has been shown that advancing the *j*th-level wavelet coefficients by $2^{j-1}\left(L-1\right)-v$, where *ν* is given by [30], [37],

(5) $$v_{LA\left(L\right)}=\{\begin{array}{ll} -L/2+1 & L=8,12,16,20;\\ -L/2 & L=10,18;\\ -L/2+2 & L=14, \end{array}$$

results in an effective zero-phase MODWT pyramid algorithm. Accordingly, features in the wavelet domain will be in alignment with those in the input signal [30], as it is shown in Fig. 3(b).

#### 3) THRESHOLD SELECTION

**FIGURE 4.**
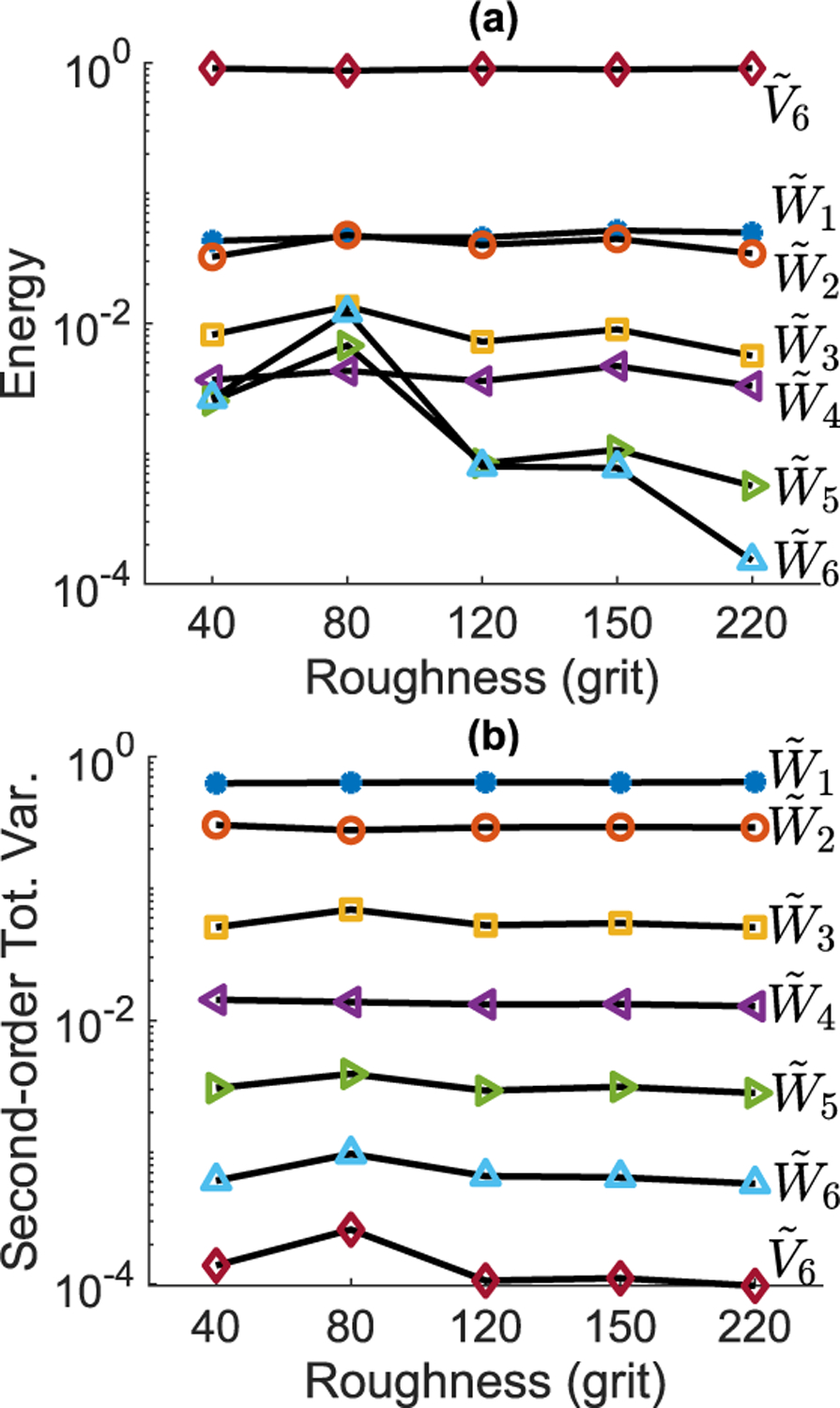
(a) The wavelet decomposition level-based energy distribution of the PABA samples with surface roughness ranging from grit 40 to grit 220, (b) the level-based second order total variation, defined by Eq. (8), for the PABA samples. Both variables are normalized to sum to unity.

In MODWT, the fine-scale wavelet coefficients, e.g. ${\tilde{W}}_{1}\left(f\right)$ and ${\tilde{W}}_{2}\left(f\right)$ when *J =* 6, mainly extract the sharp transitions in the signal. In the THz reflectivity, these transitions are either caused by the scattering-induced artifacts, which appear as noisy features with high queferency, anagram of frequency, content (spectral content of the Fourier-domain data) [21], [42], or the water vapor absorption lines, which are sharper than most solid-state materials’ resonant modes. Therefore, because the derivative operator exhibits a high-pass filter’s characteristics, the fine-scale wavelet coefficients associated with higher-queferency transitions in the derivative spectrum will have the largest magnitudes in the MODWT output. Consequently, the wavelet shrinkage techniques that use the fine-scale wavelet coefficients to specify the noise level [43] cannot retrieve the resonant signatures. Moreover, due to the recursive operation of the pyramid algorithm, this sharp transitions, gradually smoothed by the scaling filters, still would appear at higher decomposition levels. Therefore, the level-based thresholding techniques, such as the one introduced in [41], are also incapable of recovering the characteristic resonant modes. To deal with these sharp transitions in the derivative spectrum prior to wavelet shrinkage, we needed to exclude wavelet decomposition levels that are not associated with the resonant signatures. As two candidates for finding these decomposition levels, Fig. 4 compares the wavelet decomposition level-based energy distribution with the decomposition level-based second-order total variation of the PABA samples with surface roughness ranging from grit 40 to grit 220. The level-based energy distribution is calculated based on the energy of the wavelet coefficients at each level. For defining the second-order total variation, we used the first-order difference, which for the wavelet coefficients at level *j* is given by,

(6) $$D_{1,j}\left(f\right)={\tilde{W}}_{j}\left(f+\delta f\right)-{\tilde{W}}_{j}\left(f\right),$$

where *δf* represents the sampling interval of *ρ*(*f*). Therefore, the second order difference at level *j* is obtained by,

(7) $$D_{2,j}\left(f\right)=D_{1,j}\left(f+\delta f\right)-D_{1,j}\left(f\right),$$

and the decomposition level-based second-order total variation at level *j* is computed by,

(8) $$TV_{2,j}=\sum_{f=f_{1}}^{f_{2}-2\delta f}\left|D_{2,j}\left(f\right)\right|.$$

where *f*_1_ and *f*_2_ indicate the beginning and ending points of the bandwidth. Figure 4(a) illustrates that the level-based energy distribution cannot differentiate the levels of decomposition at all roughness degrees. In contrast, the second-order total variation, shown in Fig. 4(b), effectively separates different levels of decomposition, regardless of the surface roughness degree. Moreover, the TV_2,*j*_ of all PABA samples with different surface roughness degrees demonstrate similar values. We observed similar trends in the *α*-lactose monohydrate’s TV_2,*j*_ at different roughness levels. We found that removing the decomposition levels whose second order total variation accounts for more than 25% or less than 0.2% of the total second order total variation in the wavelet domain prior to wavelet shrinkage can significantly improve the results. For the remaining decomposition levels, we performed wavelet shrinkage by defining a noise and scattering interval at each level, similar to the approach proposed in [41] for the time-domain THz signals. Note that the phase corrections discussed earlier are necessary here to ensure the alignment of the features at different scales. Here, we found that using a 200-GHz spectral window centered at 300 GHz and a 200-GHz spectral window centered at 1.7 THz yields the best results in the wavelet shrinkage given by,

(9) $${\tilde{W}}_{j}\left(f\right)=\{\begin{array}{ll} {\tilde{W}}_{j}\left(f\right) & \left|{\tilde{W}}_{j}\left(f\right)\right|\geq \tau_{j}\\ 0 & \left|{\tilde{W}}_{j}\left(f\right)\right|<\tau_{j}, \end{array}$$

where *τ*_*j*_ is the amplitude of the largest wavelet coefficient in the selected intervals at level *j*. After modifying the wavelet coefficients, we implemented the inverse MODWT pyramid algorithm given by,

(10) $${\tilde{V}}_{j-1}(f)=\sum_{k=0}^{L-1}\tilde{h}(k){\tilde{W}}_{j}\left(f+2^{j-1}k\;\text{mod}\;N\right)\;\;+\sum_{k=0}^{L-1}\tilde{g}(k){\tilde{V}}_{j}\left(f+2^{j-1}k\;\text{mod}\;N\right),$$

recursively until reaching ${\tilde{V}}_{0}\left(f\right)$, which represents the final reconstructed signal. Note that because in the inverse MODWT, wavelet and scaling filters, $\tilde{h}\left(k\right)$ and $\tilde{g}\left(k\right)$, identical to those used in the decomposition algorithm are employed, it is important to reverse the circular advancements applied in the phase correction step prior to the spectral reconstruction.

## III. RESULTS

### A. SPECULAR REFLECTIVITY

**FIGURE 5.**
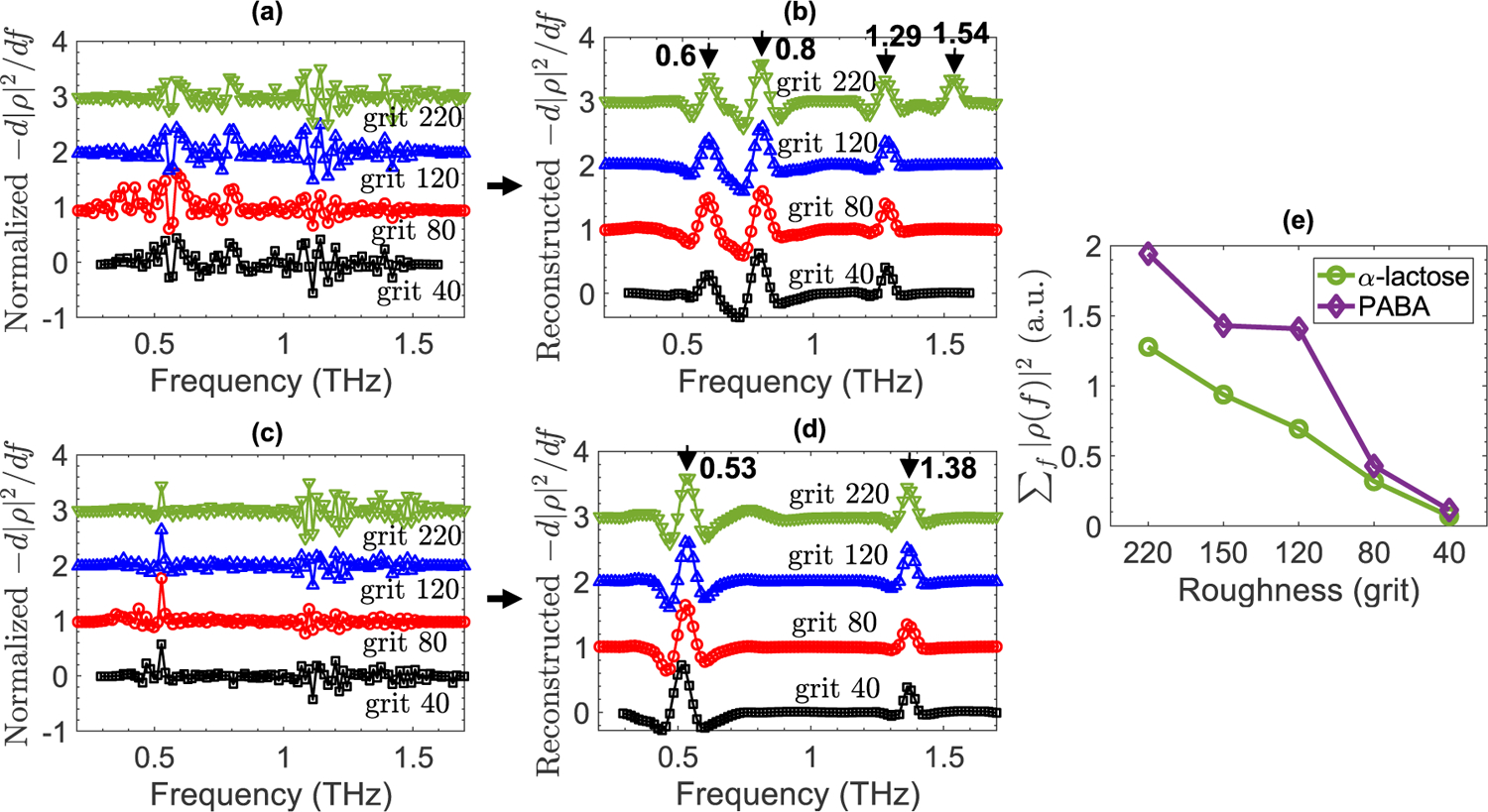
(a) The vertically-offset derivative of THz specular reflectivity from PABA samples with grit 40, 80, 120, and 220 rough surfaces, (b) wavelet shrinkage outcome for the derivative of PABA samples shown in (a), (c) the vertically-offset derivative of THz specular reflectivity from *α*-lactose samples with grit 40, 80, 120, and 220 rough surfaces, (d) wavelet shrinkage outcome for the derivative of *α*-lactose samples shown in (c), (e) the integral of the specular reflectivity (normalized THz power reflectivity) for PABA and *α*-lactose from sample pellets with grit 40, 80, 120, 150, and 220 rough surfaces.

Here, we illustrate the results of the proposed wavelet shrinkage technique in specular reflectivities of PABA and *α*-lactose monohydrate. Figure 5(a) shows the vertically-offset derivative of reflectivity from PABA samples with grit 40, 80, 120, and 220 rough surfaces. Obviously, peak recognition based on the peak height thresholding would not yield accurate resonant frequencies in the derivative spectrum. However, as shown in Fig. 5(b), in the wavelet shrinkage outcome, only the PABA’s true resonant frequencies at 0.6, 0.8, 1.29, and 1.54 THz appeared as the local maxima. Importantly, the resonant features at 1.29 and 1.54 THz were completely obscured in Fig. 5(a). Yet, they were successfully recovered in the derivative spectra reconstructed from the modified wavelet coefficients. As shown in Fig. 5(e), increasing the surface roughness level resulted in a significant drop in the available bandwidth through the specular detection angle. Therefore, the 1.54 THz resonant frequency, already close to the measurements’ noise floor, could not be resolved in samples with grit 120, 80, and 40 rough surfaces. Likewise, Fig. 5(c) illustrates the vertically-offset derivative of reflectivity from *α*-lactose monohydrate samples with grit 40, 80, 120, and 220 rough surfaces. Although peak thresholding could resolve the sharp resonant signature at 0.53 THz, *α*-lactose’s resonance at 1.38 THz was completely obscured at all roughness levels. Figure 5(d) demonstrates that in the wavelet shrinkage outcome both resonant features were clearly distinguished as local maxima, without being disturbed by other spectral artifacts. Note that the *α*-lactose’s resonant mode at 1.2 THz has a weaker amplitude and full width at half maximum (FWHM) in comparison to the resonant features at 0.53 and 1.38 THz. Moreover, wavelet coefficients associated with the 1.2 THz resonance are smaller than the threshold values set based on the highest amplitude in the noise and scattering intervals at each decomposition level. Therefore, the 1.2 THz resonant frequency could not be recovered in the reconstructed reflectivity spectra. Additionally, for both PABA and *α*-lactose samples with grit 40 rough surfaces, because of the lower SNR, we were able to apply the wavelet shrinkage in a slightly smaller spectral range.

### B. OFF-SPECULAR REFLECTIVITY

**FIGURE 6.**
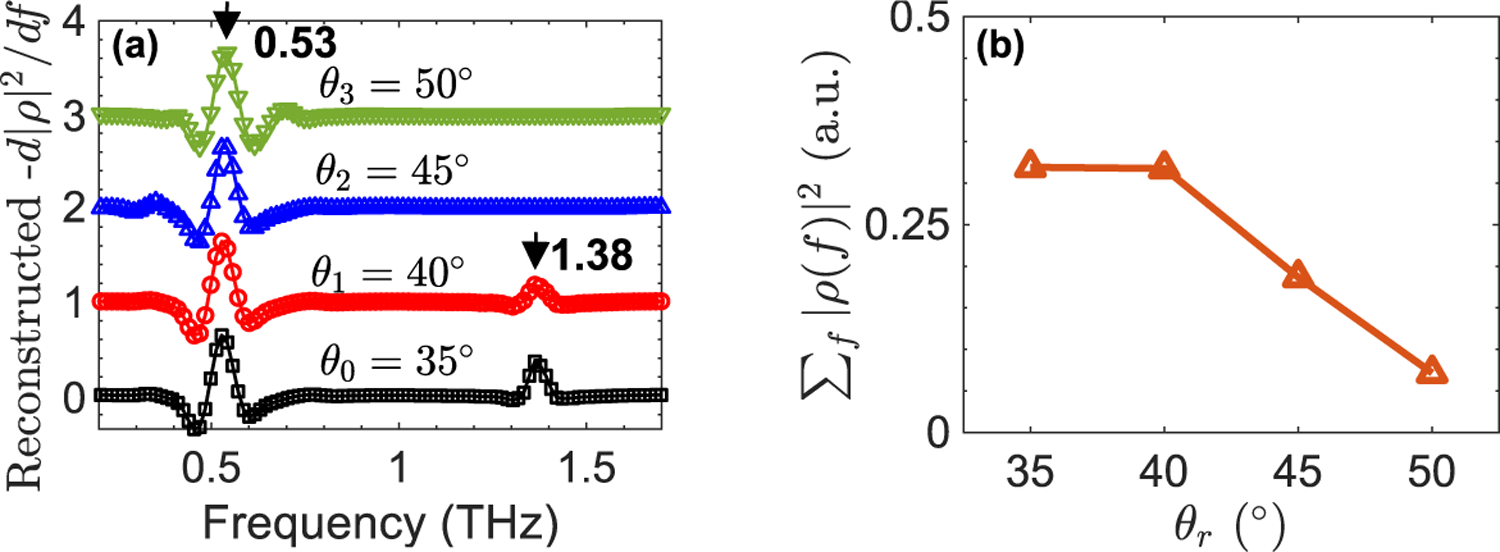
(a) The wavelet shrinkage of the derivative of reflectivities from a grit 80 rough surface *α*-lactose sample at specular and off-specular angles, including *θ*_0_ = 35°, *θ*_1_ = 40°, *θ*_2_ = 45°, and *θ*_3_ 50°. The resonant signature at 1.38 THz was only identifiable at *θ*_0_ = 35° (specular), and *θ*_1_ = 40°, (b) the integral of reflectivity of an *α*-lactose monohydrate sample with grit 80 rough surface at detection angles *θ* = 35° (specular), *θ* = 35°, *θ* = 40°, and *θ* = 45° (off-specular).

Although the off-specular THz reflectivity has been utilized for chemical recognition in crystalline solids [20], [21], it either has been averaged with the specular reflectivities [21], or uncharacteristic spectral artifacts have reduced its applicability [20]. Here, we demonstrate that the wavelet shrinkage technique is also effective for the retrieval of characteristic resonant frequencies from the derivative of THz off-specular reflectivities, despite their lower SNRs. Figure 6 shows the outcome of the wavelet shrinkage method applied to the derivative of reflectivities from a grit 80 rough surface *α*-lactose sample at *θ*_0_ = 35° (specular), *θ*_1_ = 40°, *θ*_2_ = 45°, and *θ*_3_ = 50°. It can be seen that as the detection angle increases, the resonant feature at 1.38 THz becomes smaller until it is vanished at *θ*_2_ = 45°. As shown in Fig. 6(b), increasing the detection angle, similar to increasing the surface roughness level, has significantly decreased the measurements’ bandwidth. Therefore, by increasing the detection angle to higher than 40°, the resonant frequency of *α*-lactose at 1.38 THz could not be resolved in the reconstructed spectra. However, the resonant signature at 0.53 THz is prominently featured in the reconstructed derivative spectra at all off-specular detection angles.

## IV. CONCLUSION

Although the reflection-mode THz-TDS is preferred for non-invasive material characterization, the phase ambiguity and the rough surface scattering remain the bottle-necks for successful real-world implementations. In this work, we presented the implementation of a wavelet shrinkage technique for the retrieval of characteristic resonant frequencies from the derivative of THz reflectivities measured at both specular and off-specular detection angles. Using this technique, we could reliably identify the characteristic resonant frequencies from the derivative of THz reflectivity, where the rough surface scattering effects were dominant and had obscured most of the resonant signatures. In particular, most higher-frequency resonant features extracted by this technique were not readily identifiable in the derivative spectrum, mainly because they were masked by the scattering effects. We evaluated the robustness of this technique over sample pellets made from *α*-lactose monohydrate and PABA, where controlled levels of rough surface scattering were applied to the samples using sandpapers of different grits. In particular, grit 40 and grit 80 sandpapers are considered to create extremely high levels of electromagnetic scattering. The ability of our computational technique to mitigate all levels of rough surface scattering without any *a priori* information on the sample materials and surface characteristics highlights its robustness and potential for stand-off detection applications. Future works may include the investigation of the effectiveness of this technique for retrieval of resonant signatures at higher frequencies using THz generation and detection techniques that enable even higher bandwidths. Moreover, the effects of the shape of a particular resonant signature, such as its height or the full-width at half maximum (FWHM), and the proximity of two adjacent resonant frequencies on the performance of the wavelet shrinkage technique can be further studied.

## Figures and Tables

**TABLE 1.** The RMS surface height and the Fraunhofer frequency for each sandpaper grit.

Grit	lactose *σ(µm)*	PABA *σ(µm)*	Literature *σ(µm)*	Fraunhofer freq. (GHz)
220	16	15	15 [22]	480
120	26	22	21 [36]	300
80	44	40	55 [36]	170
40	93	91	135 [21]	80
